# Multisegmented Foot and Lower Limb Kinematics During Gait in Males with Chronic Ankle Instability: Exploring Links with Hip Abductor Strength

**DOI:** 10.3390/jcm14175977

**Published:** 2025-08-24

**Authors:** Maciej Olszewski, Piotr Krężałek, Joanna Golec

**Affiliations:** 1 Doctoral School, University of Physical Culture in Kraków, 31-571 Kraków, Poland; 2 Laboratory of Biophysics and Movement Analysis, Department of Biomechanics, University of Physical Culture in Kraków, 31-571 Kraków, Poland; piotr.krezalek@awf.krakow.pl; 3 Institute of Rehabilitation in Traumatology, University of Physical Culture in Kraków, 31-571 Kraków, Poland; joanna.golec@awf.krakow.pl

**Keywords:** chronic ankle instability, multisegmented foot model, gait analysis, lower limb biomechanics, hip abductor strength

## Abstract

**Background/Objectives:** Although considerable progress has been made in understanding lateral ankle sprains (LAS) and chronic ankle instability (CAI), recurrent injury rates remain high. This highlights the need to explore additional contributors such as comprehensive lower-limb gait analysis, including multisegmented foot models and proximal joint kinematics and strength. This study aimed to assess multisegmented foot and lower-limb kinematics throughout the gait cycle in individuals with CAI compared to healthy controls. Additionally, associations between hip abductor strength and frontal plane ankle kinematics were examined. **Methods:** Fifty males (25 with CAI and 25 healthy controls) participated in this cross-sectional study. Gait analysis was conducted using a BTS SMART 3D motion capture system to assess multisegmented foot and proximal joint kinematics. Isometric hip strength was measured using a Biodex dynamometer. Statistical Parametric Mapping (SPM) was used to assess group differences, and correlations were calculated between hip abductor strength and ankle kinematics. **Results:** The CAI group demonstrated significantly greater calcaneus abduction relative to the shank in the transverse plane between 88% and 93% of the gait cycle (MD = −3.50°, 95% CI [−5.60, −1.40], *d* = −0.95, *p* = 0.037). No other statistically significant between-group differences in hip, knee, or foot segment kinematics were detected. Furthermore, correlations between hip abductor strength and ankle frontal plane kinematics were not significant. **Conclusions:** Males with CAI demonstrated altered rearfoot kinematics in the transverse plane during the terminal swing phase. The multisegmented foot model was valuable in detecting subtle deviations and emphasized the importance of including the swing phase. Hip abductor strength was not associated with ankle kinematics, suggesting that its potential role in CAI may involve other mechanisms. These findings may support clinical gait assessment and rehabilitation planning by highlighting the importance of evaluating all foot segments and the entire lower limb, rather than focusing solely on the ankle joint. Segment-specific deviations, particularly those emerging during the swing phase, may help guide targeted interventions aimed at improving foot positioning in males with CAI.

## 1. Introduction

Lateral ankle sprains (LAS) are among the most common musculoskeletal injuries, affecting over two million individuals annually in the United States alone [1]. Alarmingly, only 11% of LAS patients undergo supervised physical therapy following injury [2]. Improper or insufficient management often leads to residual impairments, with approximately 46% of LAS cases progressing to chronic ankle instability (CAI) [3], which is characterized by recurrent ankle sprains, giving-way episodes, and self-reported dysfunction [4]. Although substantial progress has been made in understanding the pathomechanics and pathophysiology of LAS and CAI, there is still no clear evidence that the incidence of recurrent injuries is decreasing. This may, in part, reflect limitations in diagnostic accuracy and suboptimal treatment strategies, highlighting the need to investigate additional factors influencing functional status and to further optimize physical therapy approaches.

Despite extensive research conducted in the CAI population, there is still a lack of detailed biomechanical studies that focus not only on the injured joint but also comprehensively evaluate the entire kinematic chain. Most studies treat the foot as a single rigid segment, thereby ignoring relevant intersegmental interactions that may contribute to functional deficits in this population. This approach is inconsistent with the complex anatomy and biomechanics of the foot, which consists of multiple articulations allowing relative movement between segments. Overlooking intersegmental foot motion can mask subtle dysfunctions in foot control, potentially leading to impaired balance, altered load distribution, and increased risk of injury recurrence. Moreover, such simplification may limit the precision of rehabilitation strategies by overlooking segment-specific impairments that require targeted interventions. Studies have shown that inversion injuries such as LAS can also damage structures beyond the ankle, including the midfoot and forefoot [5]. Søndergaard [6] demonstrated that these regions are frequently affected during inversion ankle sprains, an observation that may be underrecognized by many clinicians and researchers. To better understand foot mechanics, several multisegmented foot models have been proposed [7], typically dividing the foot–ankle complex into shank, rearfoot, midfoot, medial/lateral forefoot, and hallux. Although these models offer a more detailed representation of foot function, their application in CAI-related research has been relatively limited. To date, most studies on gait analysis in individuals with CAI have used single-segment foot models and reported differences such as increased foot inversion [8] and plantarflexion [9]. These findings may be present during terminal swing, initial contact [10,11], or throughout the entire gait cycle [12] in people with CAI compared to controls.

Only a limited number of studies have evaluated gait in individuals with CAI using a multisegmented foot model. Fraser et al. [13] found increased rearfoot inversion during midstance (from 34% to 91% of the stance phase) compared to healthy controls, with a mean difference (MD) of 3.6°. In that study, no other differences were found in foot–ankle complex segments. Dingenen et al. [14] reported decreased dorsiflexion during the loading response and a more inverted rearfoot during the initial swing phase in individuals with CAI. They also did not find any differences in the midfoot and forefoot segments. In De Ridder’s study [15], a multisegmented foot model was also compared with a rigid foot model during walking and running. The authors revealed different findings than others, specifically a more everted rigid foot position from 11% to 73% of the stance phase, while no significant differences were found for the rearfoot during walking, which contradicts findings from multiple studies [8,9]. This discrepancy may arise from the absence of normalization to reference values or differences in participants’ specific characteristics. In that study, the medial forefoot showed increased inversion (MD = 9.4°) from 87% to 98% of the stance phase in the CAI group compared to controls. In two of the above-mentioned studies [13,15], foot kinematics were analysed only during the stance phase of gait. This disregards the swing phase, which also appears to be significant for people with CAI. Previous studies [10,11] reported that individuals with CAI exhibit a more inverted foot position even before heel strike. Koldenhoven et al. [16] indicated greater hip joint adduction during the swing phase across three different walking speeds in CAI participants compared to copers, which may be linked to altered ankle positioning. In addition, Huang et al. [17] found greater frontal plane hip variability during gait in individuals with CAI compared to healthy controls. However, none of the studies using multisegmented foot models incorporated kinematic data from proximal lower limb joints, which may hinder comprehensive gait evaluation in the CAI population.

Hip abductor strength deficits and diminished isometric hip extensor strength have been confirmed by a recent meta-analysis in individuals with CAI, while no decrease in isokinetic strength was noted [18]. Similar impairments may also be present during gait and functional movements. Activity of the gluteus medius, which is the primary hip abductor, may be decreased in this population, as reported by ultrasound studies [19,20,21]. Isometric strength of the hip abductor has also been shown to predict ankle sprains in soccer players [22]. While the role of this muscle group in CAI continues to be explored, little is still known about how it influences the function of distal lower limb segments. One possible explanation may lie in observed relationships between isometric hip abductor strength and dynamic balance [23,24,25,26], which is commonly impaired in the CAI population [27]. It is also speculated that optimal hip abductor function may contribute to improved control of frontal plane ankle motion, for example by reducing excessive inversion or enhancing mediolateral postural stability [28]. Foot placement is adjusted in response to changes in the centre of mass (CoM) state, primarily through modulation of hip abductor activity during the swing phase. This control mechanism relies on sensory input, including visual, vestibular, and proprioceptive signals, and is regulated through both spinal and supraspinal pathways. Proprioceptive feedback from the hip muscles may help the central nervous system estimate hip position in the frontal plane and activate the appropriate muscles to correct deviations, supporting stable mediolateral foot placement and overall gait stability [29,30]. Holmes et al. [31] reported that greater hip abductor strength may be associated with a greater foot progression angle in healthy males, although such a relationship was not found in females. To date, no studies have focused on exploring the direct relationship between hip abductor strength and frontal plane ankle kinematics during gait. Consistent with this, our previous study found that isometric hip abductor strength was associated with self-reported instability and dynamic balance performance [23]. On this basis, this variable was selected for the correlation analysis in the present study, as such information may aid in better understanding the biomechanical role of hip musculature in individuals with CAI.

Despite promising results from existing multisegmented foot models, their limited application in CAI research, particularly without the inclusion of proximal joint kinematics and full gait cycle analysis, continues to hinder a comprehensive understanding of broader biomechanical adaptations in this population. Therefore, the primary aim of this study was to assess multisegmented foot and lower limb kinematics throughout the entire gait cycle in males with CAI, compared to healthy controls. It was hypothesised that males with CAI would demonstrate altered foot segment and proximal joint kinematics across the gait cycle, particularly increased rearfoot inversion and hip adduction relative to healthy controls. Secondarily, the study aimed to examine whether hip abductor strength is associated with ankle kinematics during gait. It was hypothesised that moderate negative correlations would be observed between hip abductor strength and both peak ankle inversion and frontal plane range of motion, indicating a potential role in limiting ankle excursion. This comprehensive evaluation may help clinicians better understand how CAI influences the biomechanics of the entire lower limb kinematic chain. The findings may support the optimisation of rehabilitation strategies for males with CAI.

## 2. Materials and Methods

### 2.1. Participants

The study enrolled 25 males with CAI and 25 healthy male controls. Participants were recruited through posters shared on social media, academic platforms, and displayed on campus. Volunteers completed an online survey to determine eligibility based on inclusion and exclusion criteria. The inclusion criteria for participants with CAI were established in accordance with the recommendations of the International Ankle Consortium [32] and included the following: (1) male sex and an age range of 18 to 30 years; (2) a history of at least one significant ankle sprain that caused pain, swelling, and activity limitation for at least one day, occurring no less than 12 months prior to study enrolment; (3) at least two episodes of the ankle “giving way” in the six months preceding participation and/or a history of recurrent ankle sprains and/or subjective sensations of ankle instability; (4) confirmed functional ankle instability, indicated by a score of ≤24 points on the Polish version of the Cumberland Ankle Instability Tool (CAIT-PL) [33]. The inclusion criteria for the control group (CON) were as follows: (1) male sex and an age between 18 and 30 years; (2) no prior history of ankle sprains; (3) no signs of functional ankle instability, confirmed by a CAIT-PL score between 28 and 30 points. Exclusion criteria for all participants included the following: (1) a history of surgeries involving musculoskeletal structures in either lower limb; (2) a history of fractures in either lower limb that required realignment; (3) any acute musculoskeletal injury to the lower limb within the three months preceding the physical assessment, affecting joint integrity or function.

### 2.2. Ethical Approval

The participants were informed about the research protocol in detail and provided their written informed consent to participate in the study. All procedures were performed in accordance with the 1964 Helsinki declaration and its later amendments. The approval of the Bioethics Committee at the Regional Medical Chamber in Krakow (No. 11/KBL/OIL/2022) was obtained for this study.

### 2.3. Study Protocol

Before the measurements, participants provided detailed information about their history of ankle sprains and instability episodes. All volunteers were asked to complete the CAIT-PL questionnaire [33] to determine the severity of CAI, as well as the Godin Leisure-Time Physical Activity Scale [34] to assess their current level of physical activity. Anthropometric data were then collected for each participant. This included measurements of body height using the Seca 213 stadiometer (Seca GmbH & Co. KG, Hamburg, Germany), body mass using the BC-418 analyser (Tanita Corporation, Tokyo, Japan), and foot posture using the Foot Posture Index [35]. For gait analysis, pelvis width, pelvis depth, lower limb length, knee width, and ankle width were measured using a measuring tape to the nearest 0.5 cm or a thickness compass to the nearest 0.1 cm. Subsequently, retroreflective markers were placed on the participant’s lower limbs according to the BTS Simple Davis protocol [36] and on the feet in accordance with the modified Rizzoli foot model [37]. All markers were applied by the same experienced examiner to ensure consistency.

Collection of kinematic data began with a static standing posture trial, which served as a reference for normalising gait data. To record lower limb kinematics during gait, participants walked barefoot at a self-selected speed along an 8 m walkway. The warm-up consisted of five minutes of walking to familiarise participants with the testing environment and ensure repeatability of the gait cycle. All participants received identical verbal instructions regarding gait performance to standardise the conditions. The mean from three consistent gait cycles was used for further kinematic and spatiotemporal analysis. Upon completion of the gait assessment, participants underwent hip strength testing. All measurements were conducted by a team of two experienced researchers.

The participant’s dominant leg was identified as the preferred leg for kicking a ball. In the CAI group, the involved limb was analysed. In cases of bilateral ankle instability, the limb with the lower CAIT-PL score and a higher number of giving-way episodes was selected. In the control group, limbs were matched prior to measurements by assigning one as “sham involved” and the other as “sham uninvolved”, which was used for subsequent analysis. This procedure ensured the same proportion of dominant and non-dominant limbs across groups, minimizing the influence of limb dominance on the results [23,38].

### 2.4. Foot Posture Index

The Foot Posture Index is a six-item scale used to assess rearfoot, midfoot, and forefoot posture in the three cardinal body planes. It was designed to quantify variation in foot position easily and quickly in a clinical setting [35]. It consists of the assessment of the following: (1) talar head palpation; (2) curves above and below the lateral malleolus; (3) inversion and eversion of the calcaneus; (4) bulge in the region of the talonavicular joint; (5) congruence of the medial longitudinal arch; and (6) abduction and adduction of the forefoot on the rearfoot (the too-many-toes sign). The scoring system uses a five-point Likert-type scale, where each item is scored from −2 to +2. The overall score is the sum of all six items. Scores between −1 and −4 indicate a supinated foot posture, while scores from −5 to −12 indicate significant supination. Values from 0 to +5 represent a neutral foot posture, scores from +6 to +9 indicate pronation, and values above +10 suggest significant pronation. The Foot Posture Index demonstrates excellent intra-rater reliability (ICC = 0.945) and moderate inter-rater reliability (ICC = 0.575) [39].

### 2.5. Kinematic Analysis

Kinematic data were collected during walking trials using a three-dimensional (3D) optoelectronic motion capture system (BTS SMART, BTS Bioengineering, Milan, Italy), equipped with six infrared cameras (sampling frequency: 70 Hz) and a piezoelectric force platform (Advanced Mechanical Technology, Inc. (AMTI), Watertown, USA). All equipment was calibrated prior to data collection [36]. The gait cycle was defined as the period from initial contact (0%) to the subsequent ipsilateral initial contact (100%). Events were manually identified with the support of synchronized force platform data.

Reflective markers were placed according to the Simple Davis Model [36] for tracking the pelvis, thigh, and shank, including anatomical landmarks on the anterior superior iliac spines, greater trochanter, lateral femoral condyle, fibular head, and lateral malleolus, as well as rigid bars placed mid-thigh and mid-shank.

Foot motion was analysed using a modified version of the Rizzoli Foot Model [37]. Markers were placed on the following landmarks of the right foot: the posterior aspect of the calcaneus, the peroneal tubercle, the sustentaculum tali, the navicular tuberosity, the bases of the 2nd and 5th metatarsals, the heads of the 1st and 5th metatarsals, and the proximal phalanx of the hallux. A modification to the original model was introduced: instead of a single marker on the 2nd metatarsal head, a virtual marker was calculated post hoc as the midpoint between the heads of the 1st and 5th metatarsals. This change was made to improve the symmetry and stability of the forefoot coordinate system. The forefoot spreads mediolaterally during stance, and individual marker placement is prone to error. Using the midpoint virtual marker provides a more reliable and symmetrical reference for defining forefoot direction and also reduces the risk of marker occlusion or overlap with the 1st metatarsal marker, particularly in participants with narrower feet or closely spaced metatarsal heads [40]. The Simple Davis and Rizzoli models were integrated at the shank segment, defined using common markers (fibular head, lateral malleolus, and mid-shank).

The following rigid segments were defined for the calculation of foot and lower limb kinematics:

- Shank: fibular head, lateral malleolus, mid-shank marker
- Foot: posterior calcaneus, 1st and 5th metatarsal heads, virtual metatarsal head
- Calcaneus: posterior calcaneus, peroneal tubercle, sustentaculum tali
- Midfoot: navicular tuberosity, 2nd and 5th metatarsal bases
- Metatarsus: 2nd metatarsal base, 1st and 5th metatarsal heads
- Toe: 1st metatarsal head, proximal phalanx of the hallux

Segment coordinate systems were defined following the convention proposed by Leardini et al. [37], with the X-axis aligned with the longitudinal axis of the segment (proximal to distal), the Z-axis oriented mediolaterally, and the Y-axis defined as orthogonal to X and Z, directed dorsally in the standing position.

Relative orientations between segments were calculated as Cardan angles using the Z–X–Y rotation sequence, corresponding to the following: Z-axis: dorsiflexion/plantarflexion, X-axis: inversion/eversion, Y-axis: adduction/abduction.

### 2.6. Hip Abductor Strength Measurement

Hip abductor isometric strength was measured using a Biodex Multi-Joint System 4 Pro dynamometer (Biodex Medical Systems, Shirley, MA, USA). Participants were positioned in a side-lying position with a strap placed around their pelvis to provide support. The isometric examination consisted of two familiarization trials at 50% and 80% of the participant’s perceived maximal torque, followed by a 30 s rest. Then, three maximal isometric contractions were performed, each lasting 5 s, with 15 s rest intervals between them [41]. The testing protocol was described in detail in our previous publication [23]. In the present study, only the isometric peak torque of the hip abductor, normalised to body mass (Nm/kg), was included as an independent variable for correlation analysis with gait-related kinematic parameters.

### 2.7. Statistical Analysis

Statistical analysis was conducted using Python 3.13 with the libraries pandas, spm1d, NumPy, SciPy, and Matplotlib version 3.10.3. For descriptive statistics of the studied population, the independent samples *t*-test (assuming normal distribution in both groups and homogeneity of variances), Welch’s *t*-test (assuming normal distribution but unequal variances), or the Mann–Whitney *U* test (when the assumption of normality was not met in at least one group) was used. The assumption of normality was verified using the Shapiro–Wilk test, and homogeneity of variances was assessed using Levene’s test.

Between-group differences in continuous kinematic waveforms were assessed using Statistical Parametric Mapping (SPM) two-sample *t*-tests. This method analyses the entire time-normalised waveform as a whole and detects continuous sections of the gait cycle where the test statistic exceeds the critical value for statistical significance, with the overall risk of type I error controlled according to Random Field Theory [42]. For each significant section, the corresponding gait time interval and *p*-value were reported, along with the mean difference (MD) in kinematic variables and the corresponding mean Cohen’s *d* effect size within that interval.

Effect sizes were interpreted according to conventional thresholds: small (|*d*| ≈ 0.2), medium (|*d*| ≈ 0.5), and large (|*d*| ≥ 0.8) [43]. Regions with medium (|*d*| ≥ 0.50) and large (|*d*| ≥ 0.80) effect sizes were shaded on the plots to indicate differences that, although not statistically significant or not meeting the predefined threshold for a large effect, may still have biomechanical relevance and serve as a basis for further research considerations.

Pearson’s correlation coefficient was used to assess linear relationships between continuous variables that followed a normal distribution. If at least one variable did not meet the normality assumption, Spearman’s rank correlation coefficient was applied instead. Correlation strength was interpreted as follows: very weak (0.0–0.2), weak (0.2–0.4), moderate (0.4–0.6), strong (0.6–0.8), and very strong (0.8–1.0).

Power analysis indicated that a minimum of 25 participants per group would be required to detect a large effect size (Cohen’s *d* = 0.80) with a statistical power of 0.80 and *α* = 0.05. This estimation was based on the large variability observed in rearfoot eversion kinematics in a multisegmented model [44]. Statistical significance was defined as *p* < 0.05.

## 3. Results

### 3.1. Characteristics of Study Population

Participant characteristics and spatiotemporal gait parameters are presented in Table 1. No significant differences were observed between the CAI and CON groups in terms of age, body mass, fat-free mass, height, BMI, or physical activity level. The CAI group demonstrated significantly lower Foot Posture Index scores, indicating a slightly more supinated static foot posture (*p* = 0.01), lower CAIT-PL scores, indicating greater functional ankle instability (*p* < 0.001), and reported a higher number of giving-way episodes in the last six months and more ankle sprains (*p* < 0.001). Spatiotemporal gait parameters, including stance phase, swing phase, stride time, and walking velocity, did not differ significantly between groups.

### 3.2. Curve Analyses

**Figure 1:**
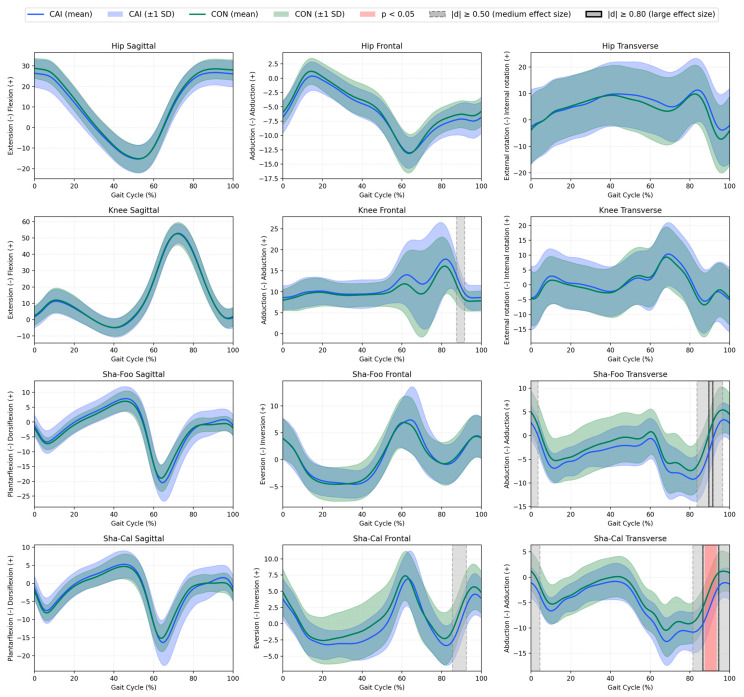
Mean kinematics (solid lines) ±1 SD (shaded areas) for the CAI group (blue) and control group (green) for the hip, knee, Sha–Foo, and Sha–Cal segments throughout the gait cycle (%). The gait cycles are time-normalized to 100%, with initial contact at 0% and toe-off at ~60%. Grey shading indicates medium effect sizes (|*d*| ≥ 0.50), black bold outline indicates large effect sizes (|*d*| ≥ 0.80), and red shading indicates statistically significant differences (*p* < 0.05) between groups, as determined by Statistical Parametric Mapping (SPM).

**Figure 2:**
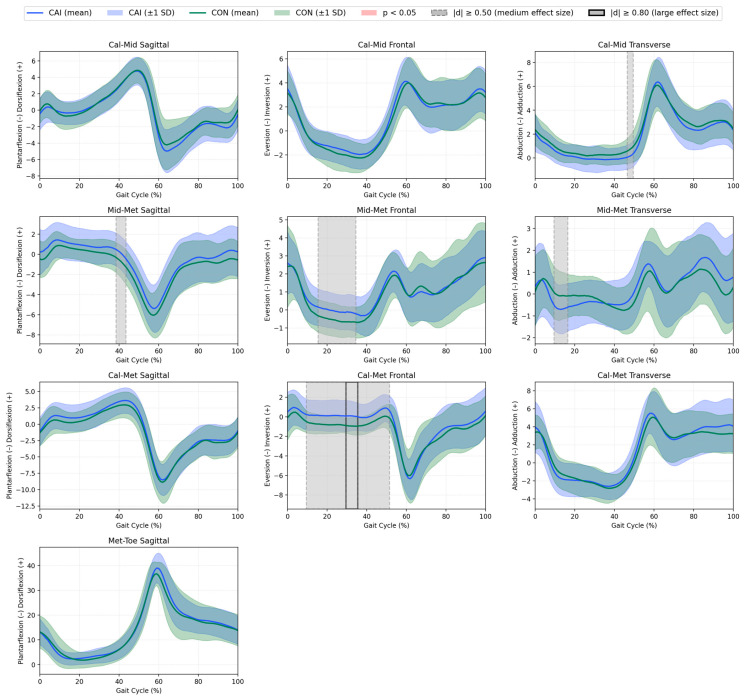
Mean kinematics (solid lines) ±1 SD (shaded areas) for the CAI group (blue) and control group (green) for the Cal–Mid, Mid–Met, Cal–Met, and Met–Toe segments throughout the gait cycle (%). The gait cycles are time-normalized to 100%, with initial contact at 0% and toe-off at ~60%. Grey shading indicates medium effect sizes (|*d*| ≥ 0.50), black bold outline indicates large effect sizes (|*d*| ≥ 0.80), and red shading indicates statistically significant differences (*p* < 0.05) between groups, as determined by Statistical Parametric Mapping (SPM).

Statistical Parametric Mapping (SPM) was used to compare the continuous kinematic waveforms between groups across the entire gait cycle (0–100%). Kinematics for all analysed joints and segments are presented in Figure 1 and Figure 2.

**Sha–Cal (calcaneus relative to the shank).** In the transverse plane, the CAI group demonstrated significantly greater calcaneus abduction than the CON group between 88% and 93% of the gait cycle (MD = −3.50°, 95% CI [−5.60, −1.40], *d* = –0.95, *p* = 0.037), as indicated by red shading in Figure 1. No other statistically significant between-group differences were observed at any time point in the frontal or sagittal planes.

**All other joints and segments**. No statistically significant between-group differences were detected in the hip, knee, Sha–Foo (foot relative to the shank), Cal–Mid (midfoot relative to the calcaneus), Mid–Met (metatarsus relative to the midfoot), Cal–Met (metatarsus relative to the calcaneus) and Met–Toe (toe relative to the metatarsus) at any time point in the sagittal, frontal, or transverse planes. Although medium (|*d*| ≥ 0.50) and large (|*d*| ≥ 0.80) effect sizes were observed in some variables (as illustrated by grey shading and black outlines in Figure 1 and Figure 2), these did not reach statistical significance in the SPM analysis.

### 3.3. Correlation Analysis

Data on hip strength in the CAI and CON groups were recently published [23]. In the present study, these variables were included solely as independent factors to examine their relationship with gait-related outcomes. No statistically significant correlations were found between hip abductor isometric strength and either the peak inversion angle or frontal plane ROM (i.e., the angular range between maximum inversion and maximum eversion) in the Sha–Foo or Sha–Cal segments during the gait cycle in either group. All observed correlations were weak or very weak (*r* ranging from −0.26 to 0.13; *p* > 0.05), with predominantly negative directions (Table 2).

## 4. Discussion

This study aimed to compare multisegmented foot and lower limb kinematics throughout the entire gait cycle between individuals with chronic ankle instability and healthy controls. Contrary to the initial hypothesis, increased inversion of the foot (Sha–Foo) or rearfoot (Sha–Cal) relative to the shank was not observed. Instead, the CAI group demonstrated greater abduction of the rearfoot during the terminal swing phase, which may reflect altered foot positioning. In addition, this study identified other non-significant but potentially interesting differences during the gait cycle as indicated by medium and large effect sizes in the knee, Sha-Foo, Sha-Cal, Cal-Mid, Mid-Met and Cal-Met. These findings may indicate that subtle alterations in segmental foot coordination in individuals with CAI are not captured by conventional single-segment foot models typically used in earlier studies. The use of a multisegmented foot model (e.g., Sha–Cal) enabled the identification of more numerous and statistically significant between-group differences in the transverse plane compared to the single-segment approach (e.g., Sha–Foo). These differences were characterised by larger effect sizes across the gait cycle, which may underscore the greater potential of multisegmented modelling in detecting nuanced kinematic deviations associated with CAI.

To date, only a few studies have examined multisegmented foot kinematics during gait in populations with CAI and compared them to healthy individuals. Fraser et al. [13] reported only increased rearfoot inversion during midstance. Dingenen et al. [14] observed decreased foot dorsiflexion during loading response and a more inverted rearfoot during the initial swing phase in individuals with CAI. De Ridder et al. [15] reported increased rigid foot eversion from 11% to 73% of the stance phase, but no differences in the rearfoot were noted during walking in CAI participants. Results of this study did not confirm previous findings indicating neither inversion [8,9,12,13] nor eversion [15] was significantly increased in the CAI group. However, a slight tendency toward increased rearfoot eversion was reported during terminal swing phase with a medium effect size but without reaching statistical significance. These discrepancies may be explained by significant differences in foot posture between the CAI and CON groups, potentially associated with greater peak rearfoot eversion and abduction in cavus feet compared to normal or planus feet [45].

Although mean Foot Posture Index (FPI) values were classified as “normal” in both groups, the CAI group presented lower FPI scores, indicating a tendency toward a more cavus/supinated foot posture. The increased Sha–Cal abduction in the CAI group occurred primarily during the terminal swing phase, a period responsible for preparing the foot for initial contact. This pattern may reflect an altered pre-positioning strategy, potentially aimed at avoiding excessive inversion upon landing. Research suggests that inappropriate foot positioning may increase susceptibility to subsequent sprains in previously sprained ankles [46]. Such compensatory abduction could represent an attempt to stabilise the ankle joint in anticipation of ground contact, but it may also contribute to lateral foot strike or uneven loading during heel contact. This pre-positioning strategy may depend on swing-phase muscle modulation in response to the mechanical state of the contralateral leg, which represents a control mechanism potentially altered in individuals with impaired neuromuscular control [47]. Biomechanically, the tibialis posterior muscle eccentrically controls excessive rearfoot eversion and abduction. Although its peak activity occurs during the stance phase [48], coordination with the peroneus longus and brevis muscles is responsible for foot alignment in the frontal and transverse planes throughout the swing phase of gait [49]. While dysfunction of the tibialis posterior may potentially be present in individuals with CAI, no studies to date have directly linked it with CAI or LAS during gait. Available research on muscle strength has only reported significant deficits in invertor concentric torque [50] and in eccentric evertor–invertor strength ratios [51].

While only the Sha–Cal segment in the transverse plane showed a statistically significant difference between groups, several other variables exhibited medium-to-large effect sizes. Although these differences did not reach statistical significance in the SPM analysis and should therefore be interpreted with caution, they may reflect subtle biomechanical deviations associated with CAI. These trends included greater knee abduction in the frontal plane, foot abduction in the transverse plane, rearfoot eversion in the frontal plane, midfoot abduction in the transverse plane, and forefoot dorsiflexion, abduction, and inversion in the sagittal, transverse, and frontal planes, respectively, in the CAI group compared to controls and were observed during both the stance and swing phases of gait. Similar alterations in forefoot kinematics have been reported by De Ridder et al. [15], who found increased medial forefoot inversion from 87% to 98% of the stance phase in individuals with CAI with a mean difference (MD) of 9.42°. In the present study, increased inversion was observed in the Mid–Met and Cal–Met segments during comparable phases of gait, albeit with smaller mean differences. Methodological differences between studies, such as dividing the forefoot into two segments rather than a single segment and the absence of static posture normalisation in the previous work, may partially explain the discrepancies in magnitude. One possible explanation for these inversion trends could involve altered muscle function, such as impaired peroneus longus activity [52,53,54], or changes in rearfoot–forefoot biomechanical coupling, which have been proposed in previous CAI research. If confirmed, such deviations could alter the position of the forefoot, potentially affecting foot loading patterns and contributing to the risk of recurrent ankle sprains, and may therefore be clinically relevant. These observations could also inform rehabilitation strategies targeting forefoot positioning and control and warrant further investigation with larger sample sizes or under different locomotor conditions.

In this study, hip and knee kinematics were also assessed alongside multisegmented foot motion to verify whether potential impairments in the foot–ankle region might be associated with alterations in proximal lower-limb joints. No significant differences were observed in proximal joint kinematics, which is consistent with the findings of most previous studies [10,11]. Increased knee abduction in the frontal plane, which was not statistically significant but demonstrated a medium effect size, was observed in the CAI group compared to controls. However, this deviation may correspond to rearfoot findings during terminal swing, as it occurred at the same point in the gait cycle. Frontal-plane knee motion may influence rearfoot positioning through lower-limb joint coupling [55]. Only the study by Koldenhoven et al. [16] reported greater hip adduction during the swing phase in individuals with CAI, possibly linked to more inverted ankle positioning at initial contact. Nevertheless, the present findings did not support this result.

The secondary aim of this study was to evaluate the relationship between hip abductor strength and ankle kinematics during walking. Although the initial hypothesis predicted moderate negative correlations between isometric hip abductor strength and both peak inversion and frontal plane range of motion in the Sha–Foo and Sha–Cal segments, it was not confirmed. Correlations ranged from very weak to weak, were predominantly negative in direction, but were not statistically significant in either the CAI or CON groups. This may suggest that no meaningful association exists between hip abductor strength and frontal plane ankle kinematics or that any potential relationship is not linear. Interestingly, in our previous study [23], we observed a significant association between hip abductor strength and self-reported instability and dynamic balance performance in individuals with CAI, which is not reflected in the current findings. Based on current scientific evidence, the role of the hip abductor in CAI may be more closely related to variable postural control strategies [28,56]. However, as this is the first study to attempt to link hip strength with ankle movement during gait in individuals with CAI, further research is warranted to verify these findings.

The results of this study have important clinical implications. Gait training interventions may improve biomechanical outcomes, such as optimising plantar pressure distribution or increasing peroneus longus activity in individuals with CAI [57]. Gait retraining may also reduce rearfoot eversion [58], particularly when associated with a decreased foot progression angle [59]. Based on our findings, exercises aimed at controlling rearfoot abduction may be beneficial. These may include swing phase control drills using visual or auditory biofeedback to improve foot positioning during gait. In addition, implementing other interventions aimed at enhancing tibialis posterior and peroneus longus function, such as resistance training, may be justified [60,61]. In this context, progressive strengthening of foot and ankle stabilisers (e.g., banded eversion and inversion exercises), as well as eccentric strengthening, endurance training, and balance activities performed on inclined platforms, where foot position sense can also be challenged and trained, may be particularly effective in improving muscle function and foot mechanics in individuals with CAI. Moreover, it has been speculated that optimal coordination between invertor and evertor muscles may influence rearfoot, midfoot, and forefoot positioning during walking [49].

The current study also has several limitations. Only males with CAI were included in order to minimise sex-specific differences in gait performance [62]. Thus, caution is warranted when generalising the results to females with CAI. Limb dominance, which may potentially affect study outcomes, was controlled by matching the dominance status of the tested limb between groups, resulting in the same proportion of dominant and non-dominant limbs in the CAI and control groups. Therefore, it was unlikely to have influenced the results. As all joint angles were normalised to a static standing trial, the observed changes represent deviations from each participant’s neutral position, rather than absolute segment orientations. Since the CAI group demonstrated significantly different foot posture compared to controls, these functional differences should be interpreted cautiously, as they may be influenced by initial alignment. Further discussion is warranted regarding when and how to normalise foot kinematic data in light of morphological variability between individuals. In the literature, angles have been reported as non-normalised values, or normalised either to a quiet standing trial or to a defined “neutral” foot position. This distinction is important, as each approach may produce different outcomes [63]. Therefore, future studies should aim to establish clear guidelines on how multisegmented foot kinematics should be presented in the CAI population. Although possible muscle activity was speculated based on kinematic patterns, electromyographic (EMG) data were not collected and marker placement reliability was not assessed, which limits direct conclusions about neuromuscular control and may affect the accuracy of kinematic measurements. Despite the fact that multisegmented foot models provide a more detailed representation of foot motion, they may also be more susceptible to measurement errors, which can constitute a limitation of this approach. Anatomical marker misplacement and soft tissue artifacts are considered the two main sources of measurement error that can affect segment coordinate systems and, consequently, joint kinematics. These errors are particularly relevant in multisegmented foot kinematics, where inter-marker distances are much smaller than in proximal body segments [40,64]. The correlation analyses between hip strength and frontal plane ankle kinematics were performed separately within each group to account for potentially different underlying mechanisms. However, they may have been underpowered to detect small-to-moderate correlations (*r* < 0.5). Therefore, these results should be interpreted with caution and confirmed in future studies with larger samples and sufficient statistical power.

## 5. Conclusions

Males with CAI demonstrated greater rearfoot abduction in the transverse plane during the terminal swing phase of the gait cycle compared to healthy controls. No significant differences were observed in in other foot segments as well as in proximal lower limb kinematics, including those of the hip and knee. The use of a multisegmented foot model may support the identification of subtle segment-to-segment interactions that could underlie functional impairments associated with CAI. The exclusion of swing phase analysis in multisegmented foot kinematics, as seen in much of the current literature, appears unjustified, as this study highlights its potential value in enhancing our understanding of foot–ankle complex function in individuals with CAI. The results of this study may support clinical practice, particularly in gait assessment and rehabilitation planning. Specifically, they encourage clinicians to consider the role of all foot segments and the entire lower limb, rather than focusing solely on the ankle joint during examination and intervention design. This broader perspective may lead to more comprehensive rehabilitation strategies that address altered foot segment coordination and improve functional gait outcomes in individuals with CAI. Lastly, no significant relationships were found between isometric hip abductor strength and frontal plane ankle kinematics during walking. This may suggest that the potential beneficial role of hip strength in individuals with CAI operates through alternative mechanisms, which should be further explored in future research.

## Figures and Tables

**Table 1:** Descriptive statistics of the studied population.

	CAI Group (*n* = 25)	CON Group (*n* = 25)	*p*-Value
Age [years]	23.1 (3.6)	21.8 (2.9)	0.139
Body mass [kg]	78.2 (12.8)	77.2 (10.3)	0.786
Body height [m]	1.81 (0.07)	1.78 (0.07)	0.153
BMI [kg/m^2^]	23.9 (3.4)	24.4 (2.7)	0.473
GODIN	60.5 (24.3)	58.6 (30.3)	0.809
Foot Posture Index	1.8 (3.8)	4.6 (3.4)	0.01 *
CAIT-PL	20 (18–22)	30 (30–30)	<0.001 *
# of ankle sprains	3 (2–4)	0 (0–0)	<0.001 *
# of ankle giving-way	2 (1–4)	0 (0–0)	<0.001 *
Spatiotemporal parameters			
Stance Phase [%]	60.0 (1.7)	59.9 (1.0)	0.934
Swing Phase [%]	40.0 (1.7)	40.1 (1.0)	0.959
Stride time [s]	1.1 (0.1)	1.1 (0.1)	0.316
Velocity [m/s]	1.2 (0.2)	1.2 (0.1)	0.900

Data are expressed as mean (SD) or median (Q_1_–Q_3_). *p*-value—probability of type I error, * statistically significant difference, SD—standard deviation, Q_1_–Q_3_—1st and 3rd quartiles, BMI—body mass index, #—number, CAIT-PL—the Polish version of the Cumberland Ankle Instability Tool, GODIN—Godin Leisure Time Physical Activity scale.

**Table 2:** Correlations between hip abductor isometric strength and ankle kinematics throughout the gait cycle.

Independent Variable	Dependent Variables								
		Sha-Foo Peak Inversion		Sha-Foo F-ROM		Sha-Cal Peak Inversion		Sha-Cal F-ROM	
		CAI	CON	CAI	CON	CAI	CON	CAI	CON
**Hip Abductor Isometric strength [Nm/kg]**	*r*	−0.09	−0.23	−0.26	−0.11	0.13	−0.19	−0.03	−0.20
	*p*	0.68	0.28	0.22	0.60	0.52	0.37	0.88	0.33

Data are expressed as Pearson’s or Spearman’s correlation coefficients (*r*). *p*—probability of type I error; Sha–Foo—foot relative to the shank; Sha–Cal—calcaneus relative to the shank; F-ROM—frontal plane range of motion; CAI—chronic ankle instability; CON—controls.

## Data Availability

The datasets used and analysed in this study are available from the corresponding author upon reasonable request.

## Abbreviations

BMI	Body Mass Index
CAI	Chronic Ankle Instability
CAIT-PL	The Polish version of the Cumberland Ankle Instability Tool
CON	Control group/Controls
CoM	Centre of Mass
Cal-Met	Metatarsus (forefoot) relative to the calcaneus (rearfoot)
*d*	Cohen’s *d* (effect size)
EMG	Electromyographic
FPI	Foot Posture Index
F-ROM	Frontal Plane Range of Motion
ICC	Intraclass Correlation Coefficient
LAS	Lateral Ankle Sprain
MD	Mean Difference
Met-Toe	Toe relative to the metatarsus
Mid-Met	Metatarsus (forefoot) relative to the midfoot
PL	Peroneus Longus
ROM	Range of Motion
Sha-Cal	Calcaneus (rearfoot) relative to the shank
Sha-Foo	Foot in relation to the shank
SD	Standard Deviation
SPM	Statistical Parametric Mapping
